# Addressing disparities in maternal health care in Pakistan: gender, class and exclusion

**DOI:** 10.1186/1471-2393-12-80

**Published:** 2012-08-07

**Authors:** Zubia Mumtaz, Sarah Salway, Laura Shanner, Shakila Zaman, Lory Laing

**Affiliations:** 1 School of Public Health, University of Alberta, 3-309 Edmonton Clinic Health Academy, 11405 – 87 Ave, Edmonton AB T6G 1C9, Canada; 2 Centre for Health and Social Care Research, Sheffield Hallam University, Montgomery House, 32 Collegiate Crescent Collegiate Campus, Sheffield S10 2BP, UK; 3 3-309 Edmonton Clinic Health Academy, 11405 – 87 Ave, Edmonton AB T6G 1C9, Canada; 4 85-K Str 77 Defence Housing Authority Lahore Cantt, Lahore, Pakistan

**Keywords:** Social exclusion, Maternal health, Gender, Caste system, Pakistan, Health care system, Class, Health policy, Pregnancy and childbirth, Antenatal care

## Abstract

**Background:**

After more than two decades of the Safe Motherhood Initiative and Millennium Development Goals aimed at reducing maternal mortality, women continue to die in childbirth at unacceptably high rates in Pakistan. While an extensive literature describes various programmatic strategies, it neglects the rigorous analysis of the reasons these strategies have been unsuccessful, especially for women living at the economic and social margins of society. A critical gap in current knowledge is a detailed understanding of the root causes of disparities in maternal health care, and in particular, how gender and class influence policy formulation and the design and delivery of maternal health care services. Taking Pakistan as a case study, this research builds upon two distinct yet interlinked conceptual approaches to understanding the phenomenon of inequity in access to maternal health care: social exclusion and health systems as social institutions.

**Methods/Design:**

This four year project consists of two interrelated modules that focus on two distinct groups of participants: (1) poor, disadvantaged women and men and (2) policy makers, program managers and health service providers. Module one will employ critical ethnography to understand the key axes of social exclusion as related to gender, class and *zaat* and how they affect women’s experiences of using maternal health care. Through health care setting observations, interviews and document review, Module two will assess policy design and delivery of maternal health services.

**Discussion:**

This research will provide theoretical advances to enhance understanding of the power dynamics of gender and class that may underlie poor women’s marginalization from health care systems in Pakistan. It will also provide empirical evidence to support formulation of maternal health care policies and health care system practices aimed at reducing disparities in maternal health care in Pakistan. Lastly, it will enhance inter-disciplinary research capacity in the emerging field of social exclusion and maternal health and help reduce social inequities and achieve the Millennium Development Goal No. 5.

## Background

Complications of pregnancy and childbirth remain the leading cause of death and disability for childbearing women in much of the developing world. Of the estimated 529,000 maternal deaths each year, 99% take place in the developing world [1]. Just thirteen countries are estimated to account for two-thirds of all these deaths. Today, the lifetime risk of death from complications of pregnancy and childbirth is one in 89 for Pakistani women, compared to 1 in 17,400 in Sweden [2].

Two decades of the Safe Motherhood initiative and inclusion of maternal mortality rate as the target for Millennium Development Goal 5 have not, to date, had a significant impact on maternal mortality rates in countries with the highest burden [3-5]. The clustering of mortality around delivery suggests that all women should have access to skilled attendance at birth and immediately after, with timely referral for emergency care if necessary. A large body of literature describes various programmatic strategies that, if put in place and accompanied by sufficient political will, funding and strengthening of the health systems infrastructure, will reduce maternal mortality [6,7]. The theme underlying these initiatives and in this literature to date is that “we know what works” and we now need to implement the interventions [6].

Missing from the published literature, however, is a rigorous analysis of *why* these simple, evidence-based, and cost-effective strategies have not been implemented. Instead, discussions rarely progress beyond acknowledging a lack of political will or the existence of inequities and systematic disparities in maternal health care services [8,9]. A critical gap in our knowledge today is a detailed understanding of the root causes of these inequities and of the political and social dynamics that reinforce these inequities. In particular, we know very little about how class and gender influence the formulation of healthcare policy, the allocation of resources, the design and delivery of maternal health services and how these factors shape disadvantaged women’s access to health services. Does the fact that maternal health services are meant for women only lead to lack of political will to provide these services? Why do some women have access to state-of-the-art care that is unavailable to others?

The present study will examine these questions in the context of Pakistani society and culture. Specifically, the proposed research will investigate (1) the ways in which disparities in access to maternal health care in Pakistan are related to gender- and class-based exclusion of specific sub-groups of the population, and (2) whether and how maternal health policies and health systems may be signalling and reinforcing larger societal values and processes of marginalization that reduce the quality of care, delay timely intervention, and/or prevent access to entirely. This research is urgently needed in Pakistan, a country characterized by poor maternal health indicators and vast inequities in availability and access to maternal health care services. Underpinned by social constructionist and critical theory approaches, the research will draw upon the “social relations” [10,11] analytical framework for understanding gender and class. The central focus of this framework is that social structures, processes, and relations create differences in the social positioning of women and men and that it is through these gendered relations that men are given a greater capacity to mobilize a variety of cultural roles and material resources to pursue their own interests. The “social relations lens” will also be used to understand why and how resources, material, human and social, may be unequally distributed between groups [12]. Knowledge transfer and policy advocacy will form important components of the research.

### Current knowledge

Maternal mortality rate is one of the few health indicators that can be rapidly and consistently decreased - almost to the point of negligibility - if appropriate actions are taken. Malaysia and Sri Lanka halved their maternal mortality rates every 6–12 years during the 1950s-1990s, a phenomenon demonstrating the importance of political will in reorganizing health services even when national GDP is relatively low [13]. But maternal health care is one of the most inequitably distributed of all health resources [14]. Survey data from 45 low- and middle-income countries indicates that even in countries with low overall levels of health care use, inequalities in skilled attendance at birth are much larger than inequalities in other types of care [15].

The present study will draw from and build upon two distinct, yet inter-related conceptual approaches to understanding the phenomenon of inequity in maternal health care services: social exclusion and health systems as social institutions.

#### Social exclusion

Disparities in maternal health care between countries are well documented. Recent evidence suggests there are equally large disparities within countries [9,14]. For example, in Chad, rich women are 23 times more likely to have a skilled attendant present during childbirth compared to poor women; in Bangladesh the difference factor is 14 [9].

A growing body of research indicates that economic poverty alone does not explain the large disparities in access to maternal health care between the rich and poor and how such inequities might best be challenged and addressed [16]. Economic poverty is relational and embedded within power hierarchies [17,18]. The concept of social exclusion may therefore be a more useful concept to draw upon to understand the disparities in access to maternal health services. Originally developed in French political discourse [19], social exclusion is a complex concept that is variably understood to range from individual ostracism or poverty to systematic societal structures that sharply define hierarchical group boundaries that serve the interests of a privileged group [20-25]. In the latter concept of exclusion, termed the *Monopoly* paradigm, social institutions such as class, political power, economic structures, and other cultural distinctions perpetuate inequality and domination [24]. In South Asia, the concept of social exclusion has been expanded by Kabeer (2006) and others to include perceptions of social identity related to caste membership [26]. In these contexts, powerful groups construct and draw upon societal beliefs, norms and values to disparage, invisibilise and demean certain groups of people, thereby justifying the denial of full rights of participation in economic, social and political life [27]. Notwithstanding the important debates around the term and concept of social exclusion [27,28], there is an emerging consensus that it is both a process and an outcome [24], and that the discrimination occurs in private social institutions such as households as well as public institutions such as the health, education and legal systems [29].

The social exclusion perspective is a useful guiding framework for the present research because it focuses attention on social relations and integrates various forms of disadvantage within a single analytic framework. The social exclusion discourse is particularly well developed in understanding the relational aspects of deprivation, how some groups are systematically denied the material and social resources that facilitate full participation in the society and the subsequent devaluation of classes thus created. It is also a useful framework to generate a greater understanding of how women in Pakistan, as a gendered group, may be socially excluded or, as some suggest, be adversely incorporated [29,30] The framework will enable us to understand, in all its complexity, how the gendered social structures, processes and relations that lead to women being systematically denied resources and full participation in society become the basis for denial of maternal health services.

#### Health systems as purveyors of social values

Historic experience from Europe and recent empirical evidence suggests health care systems are the key to reducing maternal mortality [8,30,31]. More recent, cutting-edge thinking suggests that health systems are not just mechanical structures that provide health care; they “are also purveyors of a wider set of societal norms and values” (p.143) [32]. According to Freedman, health systems should be considered “core social institutions, culturally embedded, politically contingent, and part of the very fabric of social and civic life” (pg. 22) [33]. More importantly, they are a common interface between individuals and the power structures that shape their broader society. The structures and operations of health systems may, therefore, signal and reinforce societal values and norms [33]. Neglect, abuse and exclusion within health care systems may thus essentially be a reflection of the experience of being poor and socially marginalized in that society [33,34]. Feminist bioethics scholars have long examined the dynamics of gender and western health care [35-37], often extending the analysis to matters of race [38], socioeconomic status, and related social categories. Similar inquiries regarding gender, power dynamics and health care in developing nations are still limited [39].

It is necessary, but not sufficient, for health systems to ensure that good quality maternity services are widely available; utilization of these services also involves the dynamics of users’ decision-making. A large body of literature addressing the three-delays-model [40] of maternal health care utilization in developing countries has tended to focus on the individual characteristics of women, their families and decision-making processes. This individualistic approach fails to consider how health system characteristics *per se* may shape the decision-making process, including the primary decision of whether or not to seek care at all. For example, health system characteristics such as abuse and under-the-table bribes may deter women and their families from seeking the care they need [41], yet avoidance is often regarded as a failure of the individual woman and her family to make appropriate decisions [8]. A better understanding of the ways in which health systems, as social institutions, may be systematically creating barriers to use thereby excluding particular sub-groups of the population, and how such exclusion can be challenged is urgently needed.

### Study setting: the Pakistani context

Pakistan provides an ideal opportunity to conduct a case study of social exclusion and maternal health services because it is characterized by poor maternal health indicators and vast inequities in access to maternal health care services. With a maternal mortality rate of 297/100,000 live births, it is one of the 13 countries estimated to contribute to two-thirds of all maternal deaths worldwide [1]. Overall, 39% of all births are supervised by skilled birth attendants. When use is broken down by wealth quintile, 77% of women in the highest wealth quintile report skilled birth attendance, compared to 16% of women in the lowest wealth quintile. Highly educated women are over three times more likely to be attended by skilled birth personnel than women with no education (86% vs. 27%) [3].

#### Class and gender in Pakistan

The structure of Pakistani society is acknowledged to be characterized by two separate institutionalized systems of inequality: class and gender [42,43]. Class in Pakistan is closely related to z*aat,* a multi-dimensional identifier similar to the Hindu concept of caste [44,45]. Severe inequities in the distribution of land, the primary resource, are interwoven with notions of *zaat* to create a hierarchical and feudal society. Ethnographic work from Northern Punjab shows that only members of the *Kammi zaat* work in low prestige occupations such as butcher, barber, tailor and carpenter. They are also considered “*katia”* (low class) and “*kameenee”* (low form of life) (pg.167) [44]. Individuals born in these *zaats* largely remain locked in such occupations, a division of labor that causes and reinforces differential access to and control over resources of all types, including social status [46].

Gender inequality is Pakistan is particularly acute. Gender roles are clearly demarcated; men are socially constructed as providers and women as dependents [47]. The social institution of *purdah*^2 ^[48] further demarcates boundaries between men and women and sets standards of female morality. Consequently, large differentials exist between women and men in access to resources of all types [49]. This of course does not suggest that gender roles and relationships are non-negotiable or that there is no variation. Pakistan is a large and heterogeneous country, and women’s gendered experiences and social relations are shaped by socioeconomic, ethnic and regional variations [45,50,51]. Nonetheless, the coexistence of social stratification in relation to class and gender means a woman may be disadvantaged because she is a woman, but her disadvantage is compounded if she is a woman belonging to a lower status *zaat *[44,52].

Ethnographic and survey data from Pakistan also suggest gender values and norms are tightly interwoven with maternal health-seeking behavior [44]. Moreover, the degree of adherence to these gendered norms varies by *zaat* and socio-economic class [44,51-53]. For example, gendered notions of *purdah* glorify women’s seclusion, but poor women often cannot afford to adhere to these norms when the practical needs of survival necessitate their mobility outside the home to collect water, work on the farm, or even relieve themselves in the fields. Studies show that poor women’s greater mobility outside the home renders them vulnerable to sexual exploitation by the higher status *zaat* or land-owning men [44,53]. Clearly, these issues have major implications for poor women’s ability to access maternal health services, many of which are located at considerable distance. There is an urgent need to better understand how class and gender interact to create barriers to women’s access to maternal health care.

#### Maternal health policies and services

An evolving global maternal/reproductive health discourse has resulted in formulation of three maternal health/reproductive health policies in Pakistan between 1990 and 2001 [54]. The first two policies were silent on gender and class in reproductive health services [55]. The 2001 policy does identify gender equity as one objective without expanding what this will entail. The latest National Maternal and Neonatal Strategic Framework, 2005–2015 has, however, pledged to “ensure availability of high quality maternal and neonatal health services to all, especially the poor and the disadvantaged” [56].

Despite this apparent political commitment, available evidence suggests that delivery of maternal health services in Pakistan is patchy and fragmented, of poor quality, and inequitably distributed and utilized [55,57,58]. Notwithstanding a wide network of facilities, a survey of public sector health facilities in Punjab showed that only 13% of facilities designed to provide basic emergency obstetric services were actually doing so [59]. The corresponding rate in North West Frontier Province (NWFP) is 10% [60]. The paucity of services is reflected in the very low C-section rates: 0.4% in Punjab and 0.89% in NWFP, when rates of between 5 -15% are generally deemed essential for safe childbirth [60,61].

In seeking to understand the persistently inadequate maternal health care services, research has largely focused on parameters of service delivery [58,59]. A few studies focus on poor governance [60]. More critical analyses argue that although the most recent health policy in Pakistan focuses on women’s health, it fails to recognize and address the *key* gendered obstacles to uptake of maternal health care services [61]. These include a paucity of female health care providers, resulting in part from gender hostility in employment settings [62] as well as limited education opportunities for females. Clearly, a robust discourse around class, gender and exclusion in the use of maternal health services has yet to start in Pakistan.

### Research questions and objectives

Reconceptualising health systems as social institutions that mirror the inequalities of wider systems of social exclusion is a promising avenue to enhance our understanding of failing maternal health strategies and to identify more effective approaches. To date, however, very little research has attempted to systematically describe exactly how the exclusionary processes function in relation to maternal health services. An emerging, but still slim body of literature has largely focused on documenting disparities in access to maternal health care using secondary survey data [9,16,17]. Still lacking are in-depth studies to determine who and what are responsible for these inequities. An understanding of these factors will provide policy-makers with much needed insight into addressing what can appear to be insurmountable, deep-seated inequalities reinforced by multiple hierarchies.

Drawing on the work by Freedman et al. [31] the proposed research will address these issues through two key questions. First, how are the axes of social exclusion constructed, mobilized and experienced in Pakistan, particularly in relation to class and gender divisions? Second, to what extent do these dimensions of social exclusion affect the ways in which maternal health care services are conceived, designed and delivered? How do these influence poor women’s access to, and experiences of, health services?

### Research objectives

1. *Conceptual objective:* The fundamental theoretical objective of the research is to develop a locally-informed, contextually-relevant conceptual framework to understand the construction and experience of key axes of exclusion relating to gender, class, and *zaat* in Punjab, Pakistan.

2. *Empirical objectives:* There are two empirical objectives:

 2.1. To map women’s experiences of maternal health care use, and specifically skilled attendance at birth and emergency obstetric care, as it is related to class, *zaat*, and related distinctions.

 2.2. To enhance empirical understanding of the ways in which class, *zaat*, gender and related social distinctions are reflected in maternal health policy documents and in the design and delivery of maternal health care services.

3. *Knowledge transfer objective:* Is to facilitate uptake of research findings to inform positive developments in maternal health policy, service design and care delivery.

## Methods/Design

### Overview

The data will be collected in two inter-linked modules over a four-year period.

• Module 1 will contribute to *conceptual objective 1 and empirical objective 2.1* using qualitative research methods that will draw upon the principles of critical ethnography.

• Module 2 will contribute to *empirical objective 2.2* using a qualitative assessment of the design and delivery of maternal health services.

• A coordinated set of knowledge dissemination activities spanning the entire project will address the *knowledge transfer objective 3*.

### Study site

The study site will be District Chakwal, North Punjab, Pakistan. The village ethnography in Module 1 will be conducted in a village to selected. Data for Module 2 will be collected from the health services serving the village. The selection of the site is purposive: The PI (Zubia Mumtaz) has previously conducted research in this region between 1997 and 2001.

**Module 1:*Conceptual Frameworks and Maternity Experiences in the Community****(conceptual objective 1and empirical objective 2.1)*

This module will address the following research questions:

1. What are the local, culture-specific understandings of *zaat* and class? How are these local Pakistani concepts related to more generally recognized sociological concepts of caste, hereditary occupational classes and hierarchies?

2. How do *zaat* hierarchies play out in social relationships, access to resources and opportunities (broadly defined)? How do *zaat* hierarchies interact with gender in creating social boundaries? How rigid or fluid are these social boundaries, and under what circumstances can they be challenged or manipulated in order to enhance access to resources and opportunities?

3. How do women and men describe their experiences of maternal health care usage, and specifically skilled attendance at birth and emergency obstetric care? The issues they face in navigating the health system, communicating with providers, and their satisfaction with services and care they receive will be examined.

4. If there are variations in the experiences explored in question 3, how are these variations related to class, *zaat*, and related distinctions?

#### Methods for module 1

A key challenge for describing the multidimensional class and gender systems is that deeply embedded social norms are rarely transparent to those who live within them. Therefore, we will conduct a village ethnography [63]. The ethnographic approach, with its emphasis on immersion in the research setting [64] is particularly suited to Module 1 research questions because it will allow us to develop a “thick description” [65] of the cultural understandings of *zaat*, class and gender and elicit subtleties of power and resource distribution. A critical ethnographic approach will be used, in that we will synthesize the traditional ethnographic focus on subjective meanings and beliefs of respondents with the insights gained from a broader historical and structural analysis [66]. Since respondents are unlikely to give concrete answers to direct questions about gender and caste, insights will be acquired by asking participants about maternal health and associated health care experiences as starting points. The data will be collected in four phases; insights from each phase will refine and inform the research questions and methods of the next, thus increasing focus and depth as the project progresses.

#### Phase 1

To reconnect with the villagers and gain acceptance into the community, the PI will conduct social mapping exercises, visit homes and meet with the village elders and power-brokers. Social mapping is a method most useful for producing a local perspective of the village geography and socio-economic and related power structures, depicting information that outsiders cannot see by direct observation [64]. As a participatory research method, the local informants are treated as knowledgeable experts. Four social mapping exercises will be conducted; two with 6–10 women and two with 6–10 men. Participants will be asked to draw maps of the village, the larger region, and the locations of available health services. The *zaatsbiradaris* and land-holding patterns in the village will be identified from these depictions.

#### Phase 2

Focusing on research questions 1 and 2 (Module 1), the two main methods used in this phase will be participant-observation and informal interviews [67]. Participant-observation, a key characteristic feature of the ethnographic approach, is a method of data collection in which the researcher observes the people as they go about their daily lives and participates in their activities [68]. Underlying the method is the belief that knowledge *about* a community is different from *understanding* it, and that the latter can only be determined and obtained by actually living in it [69]. This method is suited to our research question because not only are gender and class sensitive issues, they permeate all aspects of life [70]. The research team members will live in the study village and immerse themselves in the lives of the villagers by helping with daily chores and participating in community events. The team will observe and record the various activities that constitute the daily life of the villagers. Informal conversations will constitute “informal interviews”.

#### Phase 3

This phase will explore in greater depth how *zaat,* class, and gender hierarchies play out in social relationships, access to resources and social opportunities by using semi-structured in-depth interview and focus group discussion methods [69,71]. Individual and group interviews are useful methods for not only exploring people’s understandings, interpretations and experiences of *zaat*, class and gender, but also of the language they use in constructing the discourse related to these concepts [72]. Sample size in qualitative research is difficult to ascertain and specify in advance. However, considering that 12 interviews are usually enough to reach data saturation [73], we estimate we will need to conduct about 20 in-depth interviews with women and 20 with men. The respondents will represent both the rich and poor groups, and all shall have had children in the past 10 years. In addition, ten older women will be interviewed since they are often the primary decision-makers around maternal health issues [54]. Eight focus group discussions, with 6–10 participants in each, will also be held, separately for women and men [74]. Representation of all *zaats and* socio-economic groups will be ensured. By this stage, the research team will be familiar with the villagers and will purposefully select the participants. Guidelines for the interviews and focus group discussion will be based on data collected during phases 1 and 2, with reference to the research objectives.

#### Phase 4

The final stage will be a tightly focused and in-depth exploration of the complex interactions among class, *zaat,* gender, and related distinctions and how they interact with maternal health-care-seeking behavior. Six longitudinal case-studies will be conducted with pregnant women belonging predominantly to disadvantaged groups [75]. Data will be collected via multiple open ended interviews, accompanying women to any antenatal care appointments or interventions, observing deliveries and subsequent post-natal care. Husbands, mothers-in-law and other family members will also be interviewed to enrich each case study. The specific focus within these longitudinal case studies will be the experiences, perceptions, rationales and decision processes that influence choices of birth attendants, the location of delivery (home or health centre), and whether to seek emergency care if a need was identified.

Each phase builds upon the next to achieve an increasingly detailed understanding of the subtle interactions among social concepts, individual experiences, and subsequent healthcare-seeking decisions. Because individual decisions are shaped by both social and personal factors, this in-depth and multi-dimensional exploration will provide a more realistic vision of the context in which maternal health policy and health service delivery must operate.

#### Data analysis

Data will be collected using audio recorders and note-taking depending on participant consent. Since the data will be collected in *Punjabi*, the local dialect, it will be translated and transcribed by native Punjabi speakers. The PI (who understand and speaks Punjabi) will double-check all interview transcripts based on audio-tapes to verify the translation and focus on conceptual equivalence. A database of the transcribed interviews, focus groups, and observation notes will be created in Atlas-ti, a qualitative data analysis software program [76]. Using a social constructivist, interpretative approach [77], data will be coded and themes identified. Initial coding will be guided by the stated research objectives and later by additional concepts as they emerge. Data analysis will be an on-going and iterative process through all phases of data collection, as early identification will allow a fuller probing of unanticipated concepts and variables in upcoming formal or informal interviews and focus group discussions. Interpretive accuracy will be assessed by triangulation of findings across the four phases, peer debriefing within the research team and other colleagues and respondent validation [78].

**Module 2:*Provision of Maternal Health Services ****(Empirical objective 2.2)*

This module will address the following research questions, all of which are considered within the context of health care as an institution that mirrors and reinforces larger patterns of social exclusion:

1. How do women of different *zaats*, class and related distinctions experience their receipt of maternal health care, specifically childbirth and emergency obstetric care?

2. What structural and procedural barriers impede disadvantaged women’s access to timely and high-quality maternal health services?

3. How do policy-level issues and related health system elements contribute to variations in women’s maternal health care access and experiences?

#### Methods in Module 2

Drawing upon the Hulton, Mathews & Stones (2000) framework for assessing quality of maternal health services in developing countries [79], both women’s experiences of care and key aspects of care provision will be examined. Women’s experiences of maternity care will include an assessment of their impression of the adequacy of human and physical resources, their understanding of the usefulness of the procedures they undergo, the respect they are accorded, their sense of dignity, equity and the emotional support they receive. Key aspects of the provision of care assessed will include the quality of human and physical resources; the quality of their referral links and information management systems; the use of appropriate technologies in caring for women and the facility/hospital’s adherence to internationally recognized good practice [77]. System-wide and policy-level contributors to women’s experiences of maternal health care will also be explored. To ensure maximal phenomenal variation [80], both the public and private health care sectors will be included. The public sector health care services are usually used by the poor, whilst the rich tend to use private sector care. In each sector, one primary health care centre/clinic and one hospital will be assessed as case studies, for a total of four health care settings. The following specific components will be undertaken using a range of methods:

1. Analysis of documents related to maternal health practices and policies from national, provincial, district and individual health care facilities. Underlying themes, assumptions, and any inconsistencies among documents will be explored [81].

2. Observation and documentation of the facility infrastructure, both physical (e.g., whether the design of the labour room respects women’s privacy) and instrumental (e.g., numbers of staff on duty; types of services available and their quality, availability of essential drugs and supplies) using a structured checklist [81].

3. Observation of 25 moment-in-time provider-patient interactions in each of the four health care settings [82].

4. Five women who come to deliver in each hospital setting ( 10 in total) will be traced from their entry into the system until they leave the facility. This will include an observation of at least 5 births overall. The points of observation will include, but not be limited to, the provider’s language and behavior toward the patient and her family, time spent with each patient and whether her concerns are addressed. Any complications or adverse outcomes will be explored in depth, which will include interviewing the woman, her family and the hospital staff and management personnel.

5. Exit interviews with ten women who delivered in each hospital (5 routine deliveries and 5 who required emergency obstetric care; 10 interviews total).

6. In-depth interviews with five physicians, five nurses, five midwives, five “Lady Health Visitors” (outreach workers from the health centers) and five traditional birth attendants.

7. Ten in-depth interviews with senior management personnel and policymakers in the district health system and provincial department of health.

Interview guidelines will be developed for each group of respondents to ensure a systematic approach, with flexibility to pursue new avenues of inquiry as insights accumulate. Because the PI is a physician herself, professional courtesies will facilitate appropriate patient contact and access to service providers and policymakers.

#### Data analysis

The data will be analyzed as follows:

i) Documentary data analysis will involve an initial careful reading to generate preliminary, exploratory themes. These will then be used to draft a coding template containing distinct “themes” of interest, each with a number of related sub-themes. This template, after piloting, will be used to guide the systematic extraction and analysis of data from all the documents so that excerpts from the documents and contextual information are entered into the relevant sections of the template for each document. An Excel spreadsheet will be used for ease of data organization.

ii) The qualitative data will be collected using audio recorders and detailed note-taking. A database of the transcribed interviews, focus groups, and observation notes will be created and merged with data obtained in Module 1 in Atlas-ti [79]. Using a social constructivist and critical interpretative approach [77], data will be coded and categorized. The categories will be queried for patterns and insights of, for example: what is the relationship between understandings of class and *zaat* with women’s experiences of the respect they are accorded, their sense of dignity, and the emotional support they receive; what are the views and understandings of policymakers and service providers of the relationships between class, *zaat* and health care resources directed to maternal health services. Interpretive accuracy will be assessed by peer debriefing within the research team and other colleagues and respondent validation [77].

**Integration, synthesis and knowledge transfer** (*knowledge transfer objective* 3)

The ultimate goal of this research project is to improve maternal health service delivery by addressing the critical barriers and failures of political will to tailor services to the needs of marginalized populations. While policymakers are clearly open to improving services (see 1.2.2, above), there is often an inadequate understanding of the deeply rooted multi-dimensional inequities embedded within societal structures. The insights to be gained from this study are clearly needed; accordingly, stakeholders will be engaged to critically reflect on the findings-to-date and assess their applicability to policy development, health service design and service delivery throughout the entire three-year research program. Two knowledge dissemination workshops (one at the end of each module) will be conducted in addition to providing ongoing engagement with key stakeholders in the government. Initial linkages have also already been established with the National Health Policy Unit of the Ministry of Health and with a number of key maternal health care program managers. One-to-one consultations with stakeholders in various professional and governmental groups will occur throughout the research program, and input will be offered to curriculum and in-service training for health professionals. Publications will include short briefing papers, contributions to professional newsletters, and peer-reviewed publications. Due to the sensitive and contentious nature of discussions involving gender, *zaat* and related social distinctions, a key element of all knowledge transfer activities will be to encourage open and thoughtful engagement with realities that are typically uncomfortable to address.

### Ethical consideration

Ethics approval for this study has been obtained from the University of Alberta Health Research Ethics Board and the National Bioethics Board, Pakistan. Voluntary verbal consent will be obtained from all respondents in module 1 since the majority of these villagers, in particular the poor, socially excluded women and men who will have low level of education. Requests for signatures in this context are viewed with a high degree of suspicion for it indicates a legal document is being signed, over which they perceive they will have little control. Written consent will be obtained policymakers, program managers and health care providers in module 2.

## Discussion

Several outcomes of the proposed research are anticipated. First, this research will produce empirical insights as well as cutting-edge theoretical frameworks and approaches to understanding and addressing social exclusion and maternal health services. This will also include theoretical advances in understanding power dynamics of gender and class that may underlie poor women’s marginalization from health care systems in Pakistan. These advances may help shed light on similar disadvantages for women in other developing countries.

Second, empirical evidence for formulation of maternal health policies and health care system practices aimed at reducing disparities in maternal care in Pakistan will be provided, with implications for other parts of the world. The research project aims to promote reduction of maternal health disparities though innovative changes in maternal health policy, primarily by grounding the design and delivery of maternal health services in a nuanced understanding of the key axes of social exclusion experienced by women. It will also aim to improve practice of health professionals via inputs to curriculum and in-service training.

Finally the research will enhance inter-disciplinary research capacity in the emerging field of social exclusion and maternal health, both in Canada and Pakistan. The project will promote knowledge and skills transfer between Canada and Pakistan, thereby supporting a key objective of Canada’s international engagement: to deliver a visible, durable impact on the world’s key development challenges, including reducing social inequalities [83] and achieving the Millennium Development Goals.

After this three-year project has concluded, and contingent upon interest among local authorities, the research team is willing to provide advice and assist development of pilot maternal health service delivery interventions and evaluation systems based on the research findings. We also hope and anticipate that this study will become the foundation for future comparative studies in different regions of Pakistan, such as urban areas, and in other developing nations. In addition, once concepts of social exclusion are clear from the in-depth, qualitative studies of this project, we hope to able to develop context-specific, measurable indicators of social exclusion. Empirical measurement of the size of socially excluded groups in the population, and the precise relationships between social exclusion and use of available maternal health care services, will then be documented.

## Endnotes

^1^ WHO defines a health system as “all the activities whose primary purpose is to promote, restore or maintain health (WHO (2001). For the purposes of this research project, we understand health systems as both facility-based health systems as well as broader public health interventions.

^2^ The word “purdah” literally means a veil. Specifically it refers to women’s covering of the face and head in the presence of men. It also refers to a range of behaviors and practices that separate women from men. Khan (2000) argues that the veil was a compromise reached within the Muslim community between outright prohibition of women from public spheres and their full admission as equals.

## Competing interests

The authors declare that they have no competing interests.

## Authors’ contributions

ZM, SS, LL, LS conceptualized the research project. SZ is supporting the implementation of the research project and playing a key role in knowledge translation activities in Pakistan. All authors have approved the final version of the manuscript.

## Author’ information

ZM (MBBS, MPH, PhD (Public Health Medicine) (PI), is a medical doctor, with expertise in gender and reproductive health with a particular focus on women’s access to reproductive health services and inequities in reproductive health policy, design and delivery of services.

SS (PhD) is the Principal Research Fellow leading the “Inequalities, inclusion and public health” research theme within the Faculty of Health & Wellbeing at Sheffield Hallam University.

LS (PhD) University of Alberta, specialises in ethics, gender studies, reproductive health law and policy.

LL (PhD is a social epidemiologist with an interest in the social determinants of youth pregnancy and sexually transmitted diseases in disadvantaged populations.

SZ (MD, PhD). specializes in epidemiology and child health.

## Pre-publication history

The pre-publication history for this paper can be accessed here:

http://www.biomedcentral.com/1471-2393/12/80/prepub
